# Rhizosphere Microbiomes from Root Knot Nematode Non-infested Plants Suppress Nematode Infection

**DOI:** 10.1007/s00248-019-01319-5

**Published:** 2019-01-21

**Authors:** Dongmei Zhou, Hui Feng, Taruna Schuelke, Alejandro De Santiago, Qimeng Zhang, Jinfeng Zhang, Chuping Luo, Lihui Wei

**Affiliations:** 1 Institute of Plant Protection, Jiangsu Academy of Agricultural Sciences, Nanjing, 210040 China; 2 Department of Nematology, University of California, Riverside, CA 92521 USA; 3 Jiangsu Provincial Key Construction Laboratory of Probiotics Preparation, Huaiyin Institute of Technology, Huaian, 223003 China

**Keywords:** Soil bacterial community, Root knot nematodes, Biocontrol, Microbial diversity

## Abstract

Root knot nematodes (RKN, *Meloidogyne* spp.) are serious pathogens of numerous crops worldwide. Understanding the roles plant rhizosphere soil microbiome play during RKN infection is very important. The current study aims at investigating the impacts of soil microbiome on the activity of RKN. In this study, the 16S rRNA genes of the bacterial communities from nematode-infested and non-infested rhizosphere soils from four different plants were sequenced on the Illumina Hi-Seq platform. The soil microbiome effects on RKN infection were tested in a greenhouse assay. The non-infested soils had more microbial diversity than the infested soils from all plant rhizospheres, and both soil types had exclusive microbial communities. The inoculation of the microbiomes from eggplant and cucumber non-infested soils to tomato plants significantly alleviated the RKN infection, while the microbiome from infested soil showed increased the RKN infection. Furthermore, bacteria *Pseudomonas* sp. and *Bacillus* sp. were screened out from non-infested eggplant soil and exhibited biocontrol activity to RKN on tomato. Our findings suggest that microbes may regulate RKN infection in plants and are involved in biocontrol of RKN.

**Electronic supplementary material:**

The online version of this article (10.1007/s00248-019-01319-5) contains supplementary material, which is available to authorized users.

## Introduction

Root knot nematodes (RKN, *Meloidogyne* spp.) are threatening pathogens of numerous crops and cause huge damages worldwide. The relationship between RKN and soil microbes can be parasitic, symbiotic, commensalistic, and antagonistic ([21, 42]). Nematode parasitic and antagonistic bacteria have been identified from the genera *Bacillus*, *Pseudomonas*, *Streptomyces*, and *Pasteuria* [51]. Members from the listed genera either spend part of their life cycle as parasites living outside their hosts or as endosymbionts [14]. For instance, *Pasteuria penetrans* is an effective biocontrol agent of *Meloidogyne incognita*, *Meloidogyne javanica*, and other related species because of its highly specific endoparasitic ability [48, 49]. Additionally, *Bacillus* spp. have nematotoxic effects on some free-living nematodes. Several field applications confirmed *Bacillus* as promising biological agents against RKN in crops like tomato, pepper, and cucumber [32, 55]. *Pseudomonas* spp. are also active against RKNs by destroying the nematode egg mass matrix, decreasing nematode egg hatching level, and specifically activating resistance-related enzymes in terms of peroxidase (POX) and phenylalanine ammonia lyase (PAL) [33, 46].

Microbial communities have been reported to suppress RKNs [6, 35, 38, 47]. Antagonistic microorganisms had been identified from the RKN-suppressive soil with mechanisms that regulate nematode population densities. Cultivation-independent and cultivation-dependent approaches have been used in several studies to analyze the diversity of bacteria or fungi associated with the plant-parasitic nematode genera *Bursaphelenchus* [51], *Heterodera* [34, 60, 61], *Rotylenchulus* [10], and *Meloidogyne* spp. [1, 16, 19]. Cao et al. [9] found that the composition and diversity of the core microbiome associated with *M. incognita* was different according to its life stages. Although these previous studies precisely and comprehensively illustrated the involvement of microbes in antagonistic interaction with plant-parasitic nematodes, works in identifying the specific group of soil bacteria associated with the occurrence of nematodes in the field are still limited.

A recent research by Castillo et al. [11] investigated the correlations between bacterial microbiome and *Pratylenchus neglectus* and *Meloidogyne chitwoodi* in Colorado. They revealed that the abundance of α-Proteobacteria *Rhodoplanes*, *Phenylobacterium*, and *Kaistobacter* positively correlated with *M. chitwoodi*, and the abundance of Bacteroidia and γ-Proteobacteria positively correlated with *P. neglectus*. However, they also found that the abundance of *Bacillus* spp., *Arthrobacter* spp., and *Lysobacter* spp. is negatively correlated with *P. neglectus* and *M. chitwoodi*. These findings indicate that specific relationships occur between bacterial microbiome and nematode populations.

It is known that the soil environment or plant species influence the bacterial community composition [22, 24]. In the case of RKN, both infested and non-infested plants have been found in the same crop fields, suggesting a specific interaction may occur between the plant and its niche environment. We speculate that there were specific microbes in non-infested and infested soil regulating the RKN activity. For this purpose, different soils from four plants infested or not infested with RKN were sampled and compared using 16S rRNA genes of the bacterial community. The microbiomes of infested and non-infested soils were used to inoculate tomato plants to test the biological control ability. In addition, bacterial strains were isolated and screened from the microbiome of non-infested soil for their biocontrol effects on RKN.

## Materials and Methods

### Soil Sample Collection

Rhizosphere soils from four locations in Jiangsu province of China corresponding to cucumber (Huaian, N 33° 43′ 34″; E 118° 58′ 35″), tomato (Yancheng, N 33° 15′21″; E120°16′38″), eggplant (Nanjing, N 34° 12′ 33″; E 119° 03′ 31″), and bitter melon (Nanjing, N 34° 12′ 33″; E 119° 03′ 31″) were sampled in 2015. The fields chosen in this study were planted with same crops for at least 3 years and had serious RKN problems in all fields. The rhizosphere soil samples of four kinds of plants were collected after the fruits harvest. Whole plants were taken out from the soil to look at the severity of root symptoms. Infested roots had lots of galls while the non-infested roots had none or very few galls as shown in Fig. S1. Roots were shaken gently to remove the soil that not tightly attached. Rhizosphere soil was collected by using a brush to separate the soil from the root system. Three replicate rhizosphere soil samples from infested and non-infested plants per location were collected. Each replicate sample had soil from five plants. All rhizosphere soil samples were used for microbial community analyses; eggplant and cucumber rhizosphere soils were used for further greenhouse experiment and eggplant non-infested soil was used for screening of potential biological control agents against RKN.

### PCR amplification and sequencing

DNA was extracted from 0.5 g soil collected from four locations using the PowerSoil® DNA Isolation Kit (MoBio Laboratories, Carlsbad, CA, USA) following the manufacturer’s instructions. DNA samples were quantified using a NanoDrop ND-1000 spectrophotometer (NanoDrop Technologies) and diluted to a final concentration of 10 ng/μL. PCR amplification was performed with 515F and 806R barcoded primers (Table S1) to amplify the V4 region of the 16S rRNA using the following PCR conditions: the reaction mix (60 μL) contained 0.6 μmol of each primer, 200 μmol of dNTPs, 10× Ex Taq reaction buffer, and one unit of Ex Taq DNA polymerase (Takara, Shiga, Japan). PCR included 31 cycles of 94 °C for 30 s, 52 °C for 30 s, and 72 °C for 45 s in an Applied Biosystems thermal cycler (GeneAmp PCR system 2700). PCR products were purified with the QIAquick Gel Extraction Kit (Qiagen, Chatsworth, CA) and quantified using Quant-iTTM dsDNA assay on a Qubit® fluorometer. Samples were pooled contributing exactly the same amount (50 ng/μL) of DNA in the final library which was constructed with NEBNext® UltraTM DNA Library Prep Kit for Illumina® (New England Biolabs, USA) according to the manufacturer’s protocol. The final library was subjected to Hi-Seq Illumina sequencing at Guangdong Magigene Biotechnology Co., Ltd. China.

### Sequence Processing and Analysis

Primers were trimmed and raw reads were quality checked with Trimmomatic (V0.33 http://www.usadellab.org/cms/?page=trimmomatic). The resulting paired-end sequences were merged using FLASH (V1.2.11, https://ccb.jhu.edu/software/FLASH/). The merged reads were further filtered for quality with mothur using default parameters (V1.35.1 http://www.mothur.org) and concatenated into a single fasta file. The merged sequences were aligned against the GREENGENES database (http://greengenes.secondgenome.com/) and grouped into operational taxonomic units (OTU) using the usearch algorithm (V8.0.1517 http://www.drive5.com/usearch/) in Qiime 1.9 pipeline at a similarity threshold 97%. Any singletons and chimeric reads were removed from the aligned sequences by using USEARCH (http://www.drive5.com/usearch/manual/chimeraformation.html) and UCHIME (http://www.drive5.com/usearch/manual/uchimealgo.html) respectively. The final representative reads were leveraged for determining phylogeny.

Alpha and beta diversities, PCA analysis and Venn diagram were measured and generated using Phyloseq package v1.22.3) in R (V3.4.2, http://www.R-project.org/) [43]. The analysis used for multiple comparisons is Tukey’s HSD (honestly significant difference) test, cut-off is *p* < 0.05.

### Greenhouse Assay

Seeds of *Solanum lycopersicum* “moneymaker” were surface-sterilized with 20% bleach for 20 min and rinsed with ddH_2_O for five times to remove trace of bleach. The sterilized tomato seeds were sown in autoclaved soil (121 °C for 60 min, five times in total) in a growth chamber (25 °C, 16-h light and 8-h dark photoperiod) and transplanted into pots at two true-leaf stage. The pots (5 cm bottom diameter and 8 cm high) were filled with 100 g sterile sandy soil containing 90% sand and 10% organic mix and each pot contained one plant. For microbiome experiment, there were 8 pots (one plant per pot) for each treatment. For bacterial inoculation experiment, there were 11 pots (one plant per pot) for each treatment.

One week after transplanting, the rhizosphere of plants was inoculated with microbiome slurry/bacterial culture (see methods below) and a total of 500 freshly hatched J2 of *M. incognita* were added to each pot at 7 days after microbial treatment. The J2 were added by transferring 1 mL of a suspension with 100 J2/mL into five holes around the plants. The pots were arranged in a randomized block design. Plants were maintained in the greenhouse at 25 °C, at day time and 22–23 °C, at night with ambient light, and they were irrigated and fertilized as needed. Three weeks after J2 inoculation, the roots were washed free of adhering soil and weighed, and galls were counted and calculated per gram root. For egg mass assay, roots were collected and fresh weight was recorded 8 weeks after J2 inoculation. Egg masses were stained blue by submerging the roots in 1 mg/L erioglaucine for 15 min. Egg masses were evaluated by counting the stained egg masses of individual root system.

### Microbiome Preparation

Microbiome slurry was prepared according to Badri et al. [3] with minor modifications. To prepare soil slurries, 10 g soil was incubated with 100 mL Hoagland solution (Phytotechnology Laboratories, KS, USA) for 1 h on an orbital shaker. After standing for an additional 1 h, the mixture was centrifuged at 2000 rpm for 5 min. The resulting supernatant, containing suspended soil microbes, was passed through a Waterman No. 1 filter paper to remove the root debris and then was passed through a 25-μm sieve to get rid of the eggs and/or nematodes and used as the unfiltered slurry. Filter-sterilized slurries were used as controls to separate biological/microbial factors from non-biological or chemical factors. For the filter-sterilized controls, the unfiltered slurry was further centrifuged at 20,000 rpm for 15 min and then filtered through 0.22 μm filters to eliminate the soil microbes.

### Bacteria Isolation

Antagonistic bacteria were isolated from non-infested rhizosphere soil of eggplant. A blend of 10 g of soil samples and 90 mL of sodium chloride (0.9%) were mixed in a shaker at 30 °C for 15 min, and diluted to 10^−4^. Then, 100 μL of the dilution was placed on R_2_A medium (0.5 g Yeast extract, 0.5 g proteose peptone, 0.5 g casamino acids, 0.5 g glucose, 0.5 g soluble starch, 0.3 g K_2_HPO_4_, 0.05 g MgSO_4_ 7H_2_O, 0.3 g sodium pyruvate, 15 g agar per liter, pH 7.2) [41]. Bacteria of different sizes and morphological appearances were individually isolated 24 h after incubation, purified, and preserved in LB medium. The purified isolates were grown in LB medium at 200 rpm, 28 °C for 18 h, and concentrations were measured and adjusted to a concentration of 1 × 10^6^ CFU/mL by gradient dilution method. To evaluate the biological control ability of the isolates, 3-week-old tomato seedlings were root-drenched with 5 mL individual bacterial cell culture (1 × 10^6^ CFU/mL). One week after the bacteria inoculation, 500 freshly hatched J2s were inoculated to the roots and the egg masses were stained and counted at 8 weeks after J2 inoculation.

### Bacteria Characterization

Physiological and morphological characteristics of the candidate strains were identified according to Bergey’s Manual of Determinative Bacteriology. The identification of potential strains was based on partial length 16S rRNA and gyrB gene sequence analysis. The 16S rRNA and *gyrB* gene sequences were amplified using the primers 27F/1492R (5′-AGAGTTTGATCCTGGCTCAG-3′ and 5′-TACCTTGTTACGACTT-3′) and UP-1S/UP-2Sr (5′-GAAGTCATCATGACCGTTCTGCA-3′ and 5′-AGCAGGGTACGGATGTGCGAGCC-3′), respectively [18, 59] and sent to GenScript Co., Ltd. (Nanjing, China) for sequencing. The 16S rRNA and gyrB sequences were analyzed using BLAST network services at NCBI, and 16S rRNA sequence of model bacteria with high similarity (99%) were selected as the reference objects. The neighbor-joining phylogenetic trees were constructed with 1000 bootstraps by using MEGA version 5.1 [50].

### Statistical Analysis

For the greenhouse experiment, the numbers of egg masses, eggs per gram of root were compared among microbial treatments and water/MS solution control. The one-way analysis of variance with Duncan’s new multiple range test (*P* < 0.05) was applied.

## Results

### Diversity and Species Richness of Bacterial Community

A total of 755,892 high-quality sequences were obtained with a median read count per sample of 31,496 (range 17,480–45,072). The high-quality reads were clustered using > 97% sequence identity into 336,004 microbial OTUs. Low-abundance OTUs (< 5 total counts) were discarded, resulting in 316,517 OTUs.

**Fig. 1:**
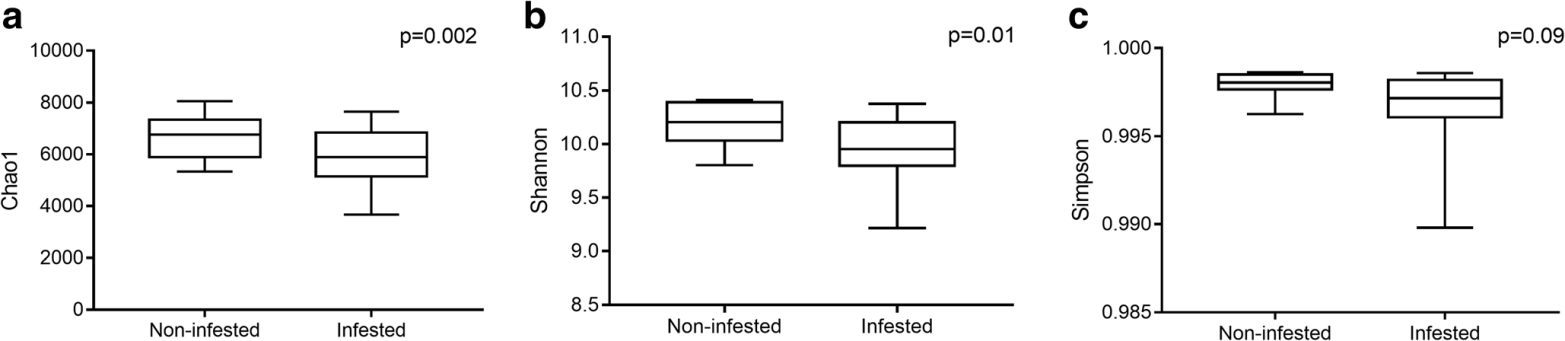
**a**–**c** Bacterial α-diversity in infested and non-infested soil samples. From left to right: the box plots are Chao1, Shannon, and Simpson indices

Alpha diversity was analyzed based on the Chao1, Shannon, and Simpson diversity indexes to assess the robustness of dataset (Fig. 1). Chao1 index reflects species richness in samples, without considering the abundance of each species. Shannon and Simpson indexes reflect the species diversity of the community [39]. The number of sequences per sample was rarefied to the minimum number of sequences in a single sample (3183 reads) before calculating the three diversity indices. As shown in Fig. 1a, the non-infested soil samples (6698 ± 797) have significant higher species richness than the infested soil samples (5891 ± 1098) measured by Chao1 index (*P* = 0.002). Additionally, it was found that the non-infested soils have higher diversity than that of infested soils (Fig. 1b, c). For the Shannon diversity and evenness estimates, there was a significant difference between the non-infested soil samples and infested soil samples (*P* = 0.01). For the Simpson diversity index, the value of non-infested soil samples was higher than that of infested soil samples (*P* = 0.09). All three measures of within sample diversity display that non-infested samples tend to contain more diverse microbial populations than infested samples.

**Fig. 2:**
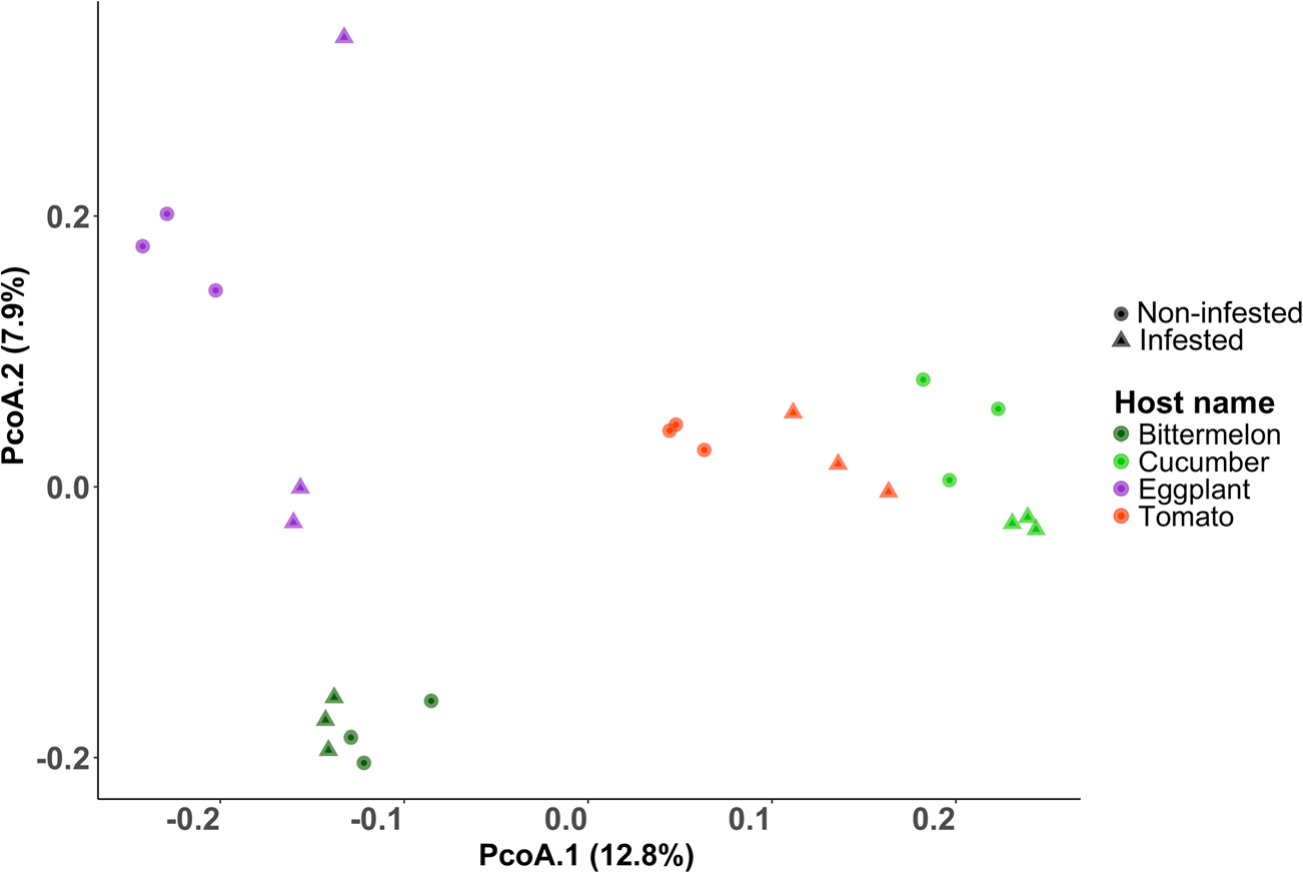
Principle coordinates analysis (PCoA) of pairwise community dissimilarities (Bray–Curtis index) based on rarefaction to 3183 sequences per sample. OTUs differentiating based on the plant type and soil type

The Bray–Curtis distances between samples were visualized with a principal coordinate analysis (PCoA). As shown in Fig. 2, samples from the same host plants clustered together based on infestation states. Moreover, samples formed distinct clusters according to host type, indicating the largest source of variation in microbial communities is due to the host plant.

### Dominant Phyla and Variation of Bacterial Community

**Fig. 3:**
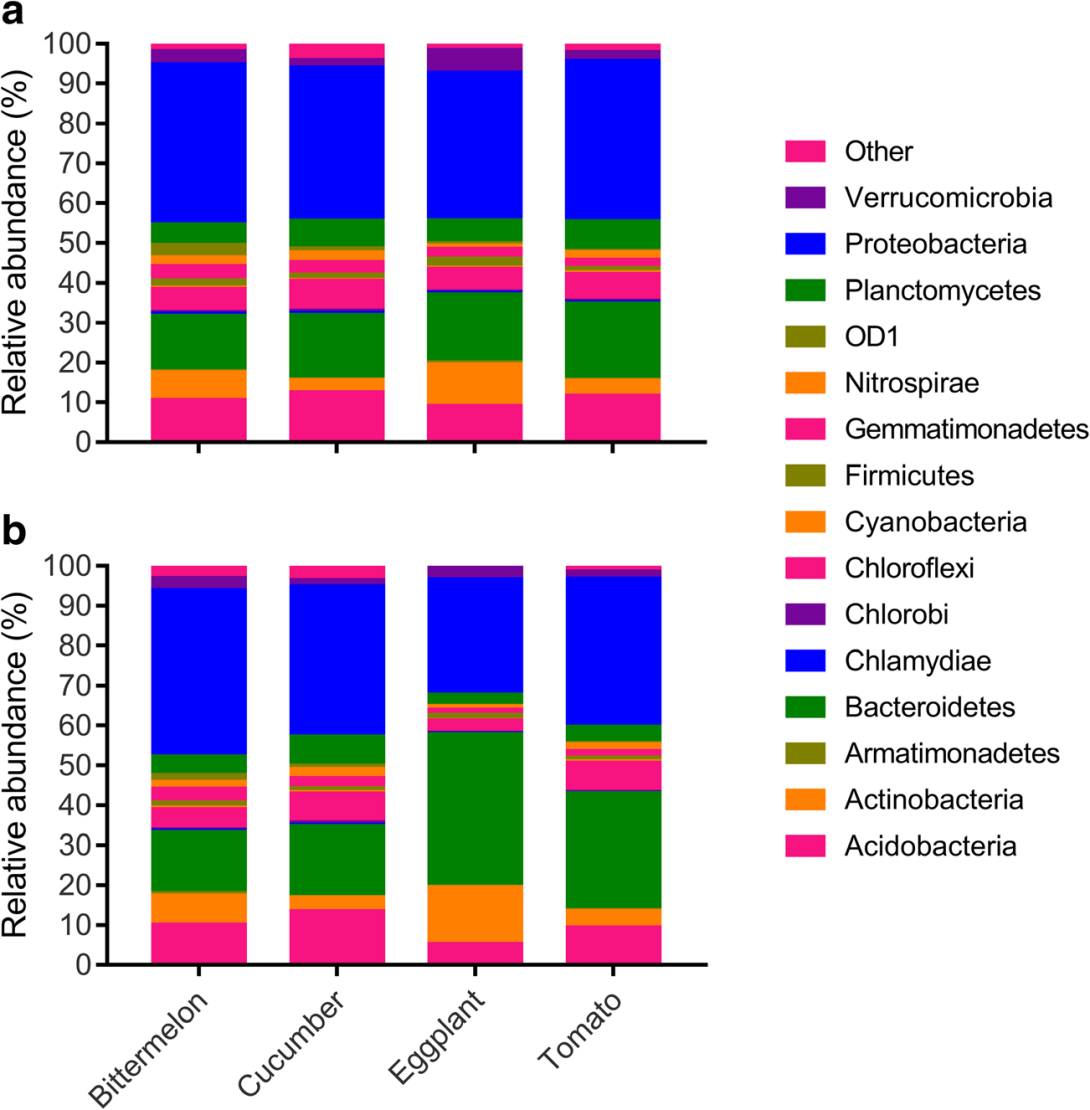
Relative sequence abundance of bacterial phyla associated with the rhizosphere soil of different plant host and infestation conditions. Major contributing phyla (top 15) are displayed in different colors. **a** Non-infested soil samples. **b** Infested soil samples

In general, Proteobacteria, Bacteroidetes, Acidobacteria, Actinobacteria, and Chloroflexi were the major phyla associated with all rhizosphere soils (Fig. 3a, b). The Proteobacteria was the most abundant, accounting for 28.9–41.2% of the total bacterial taxa in all rhizosphere samples. The Bacteroidetes was the second abundant phylum which accounted for 15.3%–38.4% of all bacterial taxa. The Acidobacteria and Chloroflexi represent about 22.7% of the total bacterial taxa in different rhizosphere samples.

The relative abundance of each phylum varied between non-infested and infested soils (Fig. S2). In general, the non-infested soils had higher relative abundance of Proteobacteria, Verrucomicrobia, Gemmatimonadetes, Nitrospirae, and Firmicutes than infested soils and had lower abundance of Bacteroidetes than infested soils. For example, the average relative abundance of Gemmatimonadetes was 26% higher in non-infested soils than in the infested soils. The average relative abundance of Firmicutes was more abundant (45% higher) in non-infested soils than infested soils. In contrast, the average relative abundance of Bacteroidetes was 46.8% higher in infested soils than in non-infested soils. Other phyla, such as Planctomycetes and Actinobacteria showed differences in soils from different plants. The infested soils from bitter melon and cucumber had higher abundance of Planctomycetes than in the non-infested soils, while the infested soils form eggplant and tomato showed lower abundance than the non-infested soils. Actinobacteria from bitter melon, eggplant, and tomato was highly abundant in the infested soil than in non-infested soil; nevertheless, Actinobacteria from cucumber was lower in infested soil than in non-infested soil. Chlorobi only showed higher abundance in infested cucumber soil. Chlamydiae were highly abundant in non-infested soils from cucumber and tomato than infested soils. OD1 had more abundance in non-infested soils from bitter melon and eggplant than in infested soils.

### Unique Taxa in Non-infested and Infested Soil

**Fig. 4:**
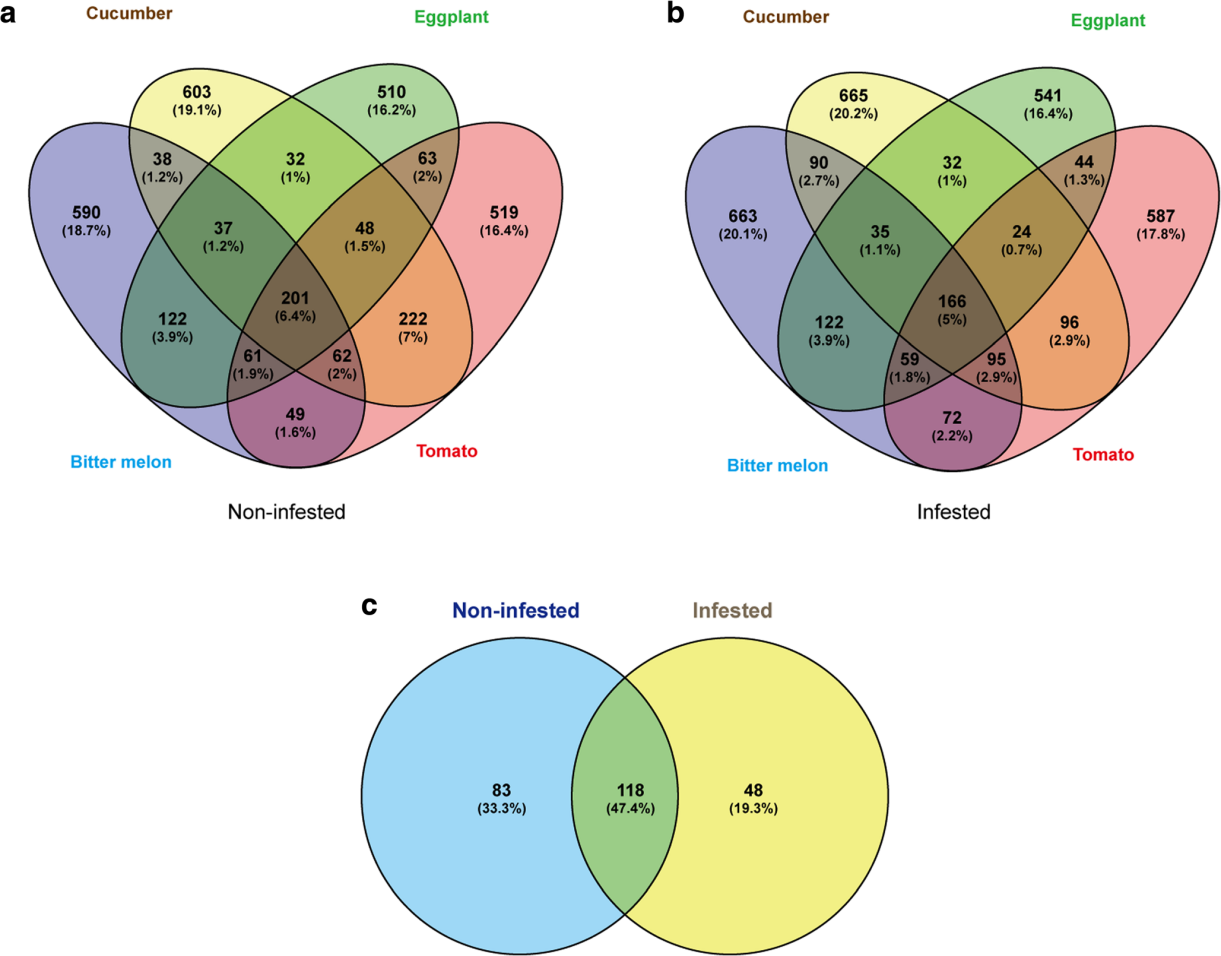
Venn diagram showing distribution of OTUs among different soil samples. **a** 201 OTUs shared across all the non-infested soil samples. **b** 166 OTUs shared across all the infested soil samples. **c** 118 OTUs shared across the non-infested and infested soil, 83 OTUs uniquely found in non-infested soil and 48 OTUs uniquely found in infested soil. Classifications of the shared OTUs are shown in Table S1

**Fig. 5:**
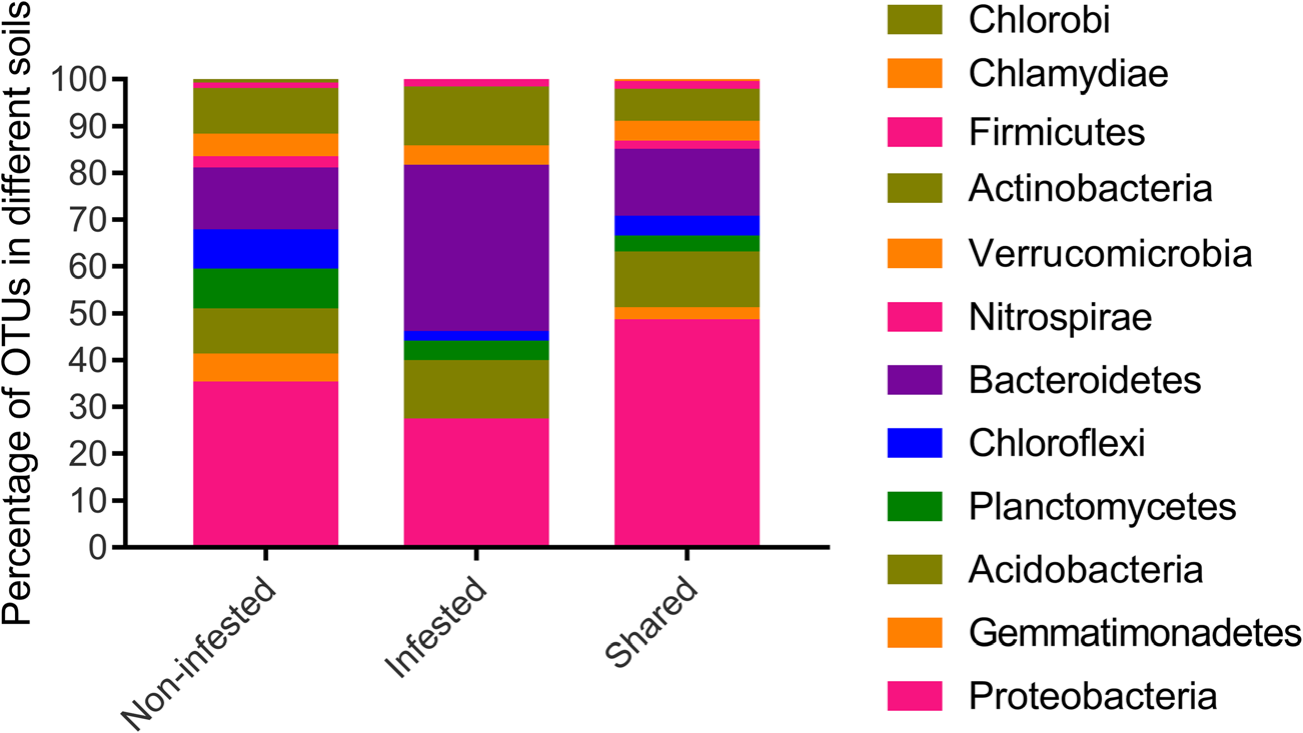
The composition of the shared OTUs in non-infested and infested soils. Twelve phyla were found in the shared OTUs of infested and non-infested soils

To identify specific phylum that were unique to non-infested or infested soil, a Venn diagram was constructed (Fig. 4). The number of the OTUs that shared in all non-infested soils was 201(Fig. 4a). The shared OTUs in infested soils were 166 (Fig. 4b). When comparing the common OTUs in non-infested and infested soils, 83 OTUs were found exclusively in non-infested soils, while 48 unique OTUs in infested soils (Fig. 4c and Table S2). Moreover, there were 118 OTUs shared in both non-infested and infested soils. Out of the 118 shared OTUs, 45 belonged to Proteobacteria (48.3%), 20 belonged to Bacteroidetes (14.4%), and 14 belonged to Acidobacteria (11.9%) (Fig. 5).

The proportions of the unique OTUs were different in non-infested and infested soils (Fig. 5 and Table 1). The Proteobacteria accounted for the most proportions in non-infested soils (34.9%) and the second in infested soils (27.1%). As shown in Table 1, at the class level, in non-infested soils, there were four classes of Proteobacteria including Alphaproteobacteria, Betaproteobacteria, Gammaproteobacteria, and Deltaproteobacteria. However, in infested soils, the Deltaproteobacteria class was not found. The orders of Pseudomonadales, Alteromonadales, Myxococcales, Rhodobacterales, Enterobacteriales, NB1-j, and MND1 were only present in non-infested soils while the orders of Caulobacterales and SC-I-84 were present in infested soils (Table S2).

**Table 1:** Classification and number of the unique OTUs found in non-infested and infested soils

Phylum	Class	Non-infested (83)^a^	Infested (48)^b^
Proteobacteria	Alphaproteobacteria	10	7
	Betaproteobacteria	6	4
	Gammaproteobacteria	9	2
	Deltaproteobacteria	4	0
Bacteroidetes	Saprospirae	5	9
	Cytophagia	3	4
	Flavobacteriia	2	3
	Sphingobacteriia	1	1
Actinobacteria	Thermoleophilia	4	0
	Acidimicrobiia	1	1
	Actinobacteria	3	4
	MB-A2-108	0	1
Acidobacteria	Acidobacteria-6	4	3
	Chloracidobacteria	4	2
	Unclassified	0	1
Chlorobi	–	1	0
Nitrospirae	–	2	0
Chloroflexi	Anaerolineae	3	1
	Gitt-GS-136	1	0
	S085	2	0
	TK17	1	0
Planctomycetes	Planctomycetia	6	1
	Phycisphaerae	0	1
	OM190	1	0
Verrucomicrobia	Opitutae	2	1
	Pedosphaerae	1	0
	Verrucomicrobiae	1	1
Gemmatimonadetes	Gemm-1	3	0
	Gemmatimonadetes	1	0
	Unclassified	1	0
Firmicutes	Clostridia	1	0
	Bacilli	0	1

^a^Number of specific OTUs from a total of 201 shared OTUs from non-infested soils planted to bitter melon, cucumber, eggplant, and tomato (cf Fig. 4)

^b^Number of specific OTUs from a total of 116 shared OTUs from infested soils planted to bitter melon, cucumber, eggplant, and tomato (cf Fig. 4)

The Bacteroidetes accounted for 13.4% in non-infested soils and 35.4% in infested soils (Fig. 5 and Table 1). There were 11 OTUs of Bacteroidetes in non-infested soils, five of them were sub-grouped into Saprospirae (Saprospirales), three of them were Cytophagia (Cytophagales), two were Flavobacteriia (Flavobacteriales), and one Sphingobacteriia (Sphingobacteriales). In comparison to non-infested soils, infested soils had 17 OTUs of Bacteroidetes, nine of them were sub-grouped into Saprospirae (Saprospirales), four were Cytophagia (Cytophagales), three were Flavobacteriia (Flavobacteriales), and one Sphingobacteriia (Sphingobacteriales) (Table S2).

Furthermore, other phyla including the Chloroflexi, Planctomycetes, Gemmatimonadetes, Nitrospirae, and Chlorobi were differently present in soil samples (Fig. 5 and Table 1). For example, there were seven OTUs which belonged to Chloroflexi and sub-assigned to Anaerolineae, S085, Gitt-GS-136, and TK17 in non-infested soils and only one OTU of Anaerolineae in infested soils. For the Planctomycetes, there were seven OTUs designated to Planctomycetia and OM190 in non-infested soils while there were two OTUs designated to Planctomycetia and Phycisphaerae in infested soils. There were five OTUs of Gemmatimonadete, two OTUs of Nitrospirae, and one OTU of Chlorobi particularly present in non-infested soils (Table S2).

### Impact of Soil Microbiomes on RKN Infection

**Fig. 6:**
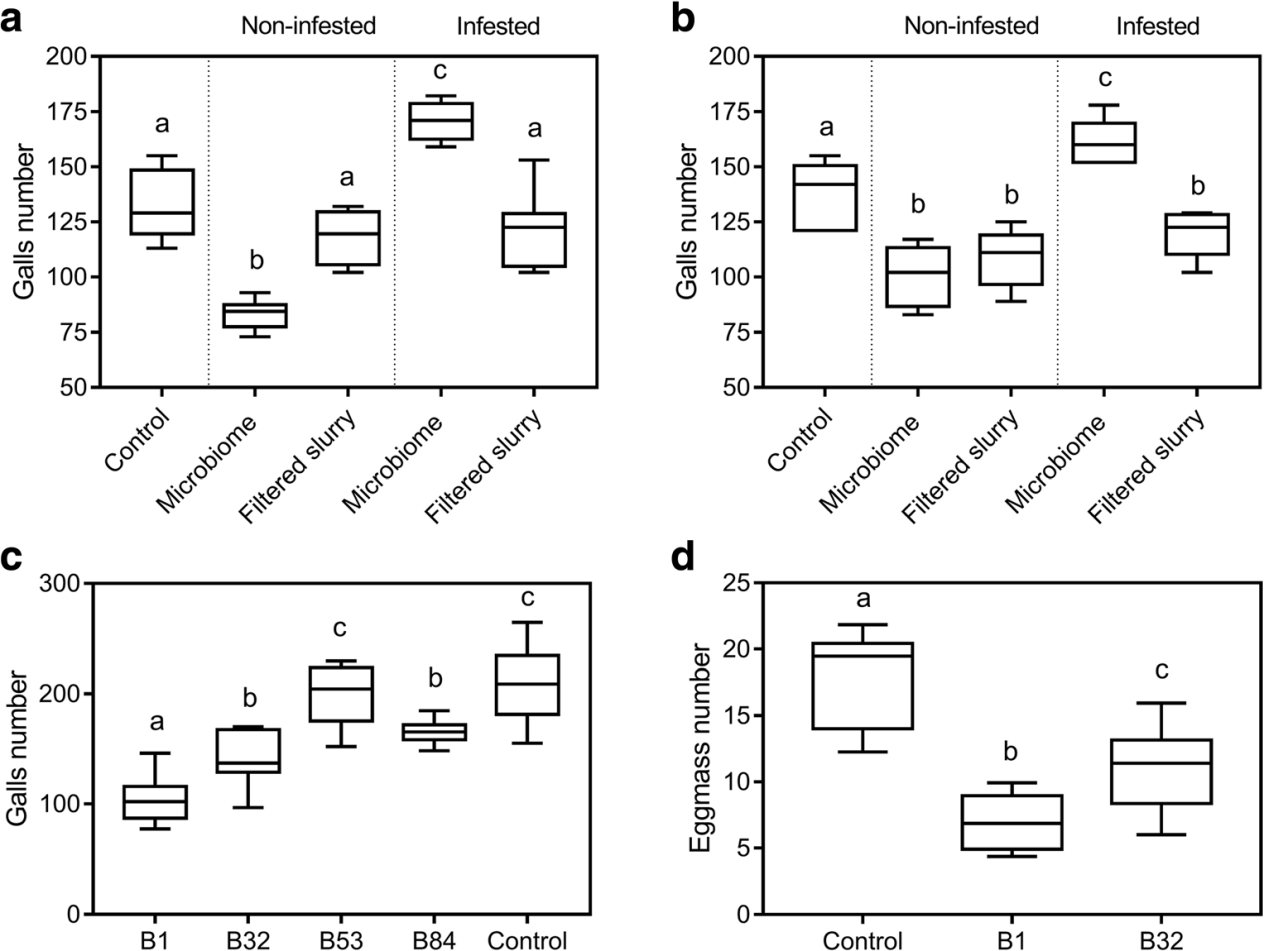
Microbial effect on root knot nematode infection. **a**, **b** Effect of soil microbiome of healthy and diseased rhizosphere soil from eggplant and cucumber on RKN infection in tomato. **c** Screening of bacterial strains against RKN infection. **d** Effect of two bacterial biocontrol strains on RKN infection in tomato. For microbiome experiments, *n* = 8; for bacterial inoculation experiments, *n* = 11. The experiments were repeated twice with similar results. One-way ANOVA was conducted followed with Duncan’s new multiple range test (*P* < 0.05) to compare the difference in RKN infection with control and different microbial treatments. Significant differences (*P* < 0.05) across microbial treatments are indicated with lowercase letters

To examine the microbiome effect on RKN infection, we counted the RKN galls in tomato plants between non-filtered and filtered treatments of eggplant and cucumber soil slurries (Fig. 6a, b). The addition of non-infested soil microbiome from eggplant and cucumber had significantly fewer galls than the control plants which treated with the same amount of MS solutions, with decreases of 38.5% and 40.2% respectively. The non-infested filtered slurry from eggplant had no difference on galls comparing with control plants, while the non-infested filtered slurry from cucumber showed lower galls than control. In contrast, tomato roots inoculated with infested soil microbiome from eggplant and cucumber had more galls than control plants, with increases of 30.8% and 17.9% respectively. The infested filtered slurry from eggplant had no difference on galls comparing with control plants, while the infested filtered slurry from cucumber reduced the galls when compared with control plants. These data showed that soil microbiomes from both infested and non-infested soil impact RKN infection significantly.

### Screening of Antagonistic Bacterial Strains Against RKN

The non-infested rhizosphere soil of eggplant was further selected to isolate the potential biological control agents against nematode infection. In total, 113 bacterial isolates were isolated and 4 isolates were used for greenhouse experiment according to their protease and chitinase enzyme activities ([56]; data not shown). The four isolates had different effects on RKN galls in tomato. B1, B32, and B84 showed significantly lower galls than untreated plants, while B53 showed no difference (Fig. 6c). B1 and B32 were selected for further greenhouse experiment to confirm their biocontrol efficacy. As shown in Fig. 6d, the tomato plants pre-treated with the B1 and B32 had significantly low egg masses than untreated control plants. Notably, the B1 lowered the egg masses at a rate of 60%. Based on the 16S rRNA and *gyrB* gene sequencing and NCBI blast, they were characterized as *Pseudomonas* sp. (B1) and *Bacillus* sp. (B32) (Fig. S3).

## Discussion

### Microbial Community and Diversity Respond to the Nematode Infection

According to the microbiome analysis, the phyla Proteobacteria, Bacteroidetes, Acidobacteria, Actinobacteria, and Chloroflex were dominant in all soil samples. This is not surprising because these five phyla have been reported as dominant bacterial communities in the rhizosphere of plants including maize, oat, cactus, potato, sugar beet, oak, and *Arabidopsis* [29, 54]. Microbial diversity is an excellent indicator of soil health [7, 31]. Loss of soil microbial diversity contributes to an increase in soil-borne plant diseases [17, 30]. The high microbial as well as functional diversity and activity are involved in plant growth promotion, plant defense, and soil-borne disease suppression [25, 26, 40]. In this study, we found that non-infested soils had higher diversity than infested soils. This indicates that the lower microbial diversity may result in high RKN infection. The scientific community has a great interest in developing strategies that reshape the rhizosphere microbial community to attain stable and more diverse conditions to prevent or mitigate pathogen/pest occurrence. Thus, knowing what kind of microbes and the role they play is very important.

In our study, the abundance and diversity of several phyla were different between non-infested and infested soils. We also found that bacteria of the phyla Proteobacteria, Verrucomicrobia, Firmicutes, Nitrospirae, Gemmatimonadetes, and other phyla were more abundant and commonly present in non-infested soils than in infested soils. Moreover, some of the OTUs were uniquely presented in non-infested soils. The Deltaproteobacteria of order Myxococcales uniquely existed in non-infested soils. The Myxobacteria has broad biocontrol properties against fungal and bacterial pathogens [8, 15] by producing antibiotics and lytic enzymes. However, to date, there are no reports of the interaction between Myxobacteria and RKN in literature.

Interestingly, five OTUs of Gemmatimonadetes were present in all non-infested soils while absent in infested soils. The phylum Gemmatimonadetes had been reported to contain phototrophic members and is poorly understood with only a handful of cultured species [62]. To date, few studies described the association of Gemmatimonadetes with nematodes. For example, Gemmatimonadetes was mainly detected on J2 of *Meloidogyne hapla* from three arable soils with nematode suppressiveness [1] and also found associated with pine wood nematode *Bursaphelenchus xylophilus* and *Bursaphelenchus mucronatus* [58]. The interaction between Gemmatimonadetes and nematodes has not been studied as far as we know; functional experiments including chemical and bioactivity studies are needed to be done on the Gemmatimonadetes.

Bacteria such as *Bacillus* spp. from Firmicutes are frequently reported as biocontrol agents against soil-borne pathogens and nematodes, with the ability to produce several of lytic enzymes and antimicrobial compounds [13, 32, 63]. Our screening results confirmed that a *Bacillus* sp. strain functions as a biological control agent against RKN.

The abundance of phylum Bacteroidetes was observed higher in infested soils than in non-infested soils. Bacteroidetes is one of the dominant phyla in soil microbiomes [29, 54] but nothing is known about its association with RKN infection.

Visualization of the Bray–Curtis distances using PCoA showed that samples formed distinct clusters based on host type. It is well-known that the rhizosphere microbiome is generally regulated by root exudates [3, 12, 20, 24]. The qualitative and quantitative composition of root exudates differs from the plant cultivar, plant species, plant developmental stage, and various environmental factors [5], which result in a certain degree of specificity in the rhizosphere bacterial community for each plant species. In this study, we had four different vegetable plants from Solanaceae and Cucurbitaceae and at different stages of infection. We found 118 shared OTUs present both in infested and non-infested soils which may be more related to plant growth and development.

### Beneficial Microbial Interaction with Plants

Studies showed that soil microbiome could induce the metabolite profile and transcriptomic changes in *Arabidopsis* and increase resistance against pathogens and insects’ infestation [4, 37, 52]. Badri et al. [4] showed that soil microbiomes potentially impacted the metabolomics profile of *Arabidopsis* and negatively influenced *Trichoplusia ni* larvae feeding. *Trichoderma gamsii* was isolated from *Arabidopsis* soil affected the feeding behavior of *T. ni* through the modification of the leaf metabolome and phytohormornes [64]. Other works showed that application of the microbiome from a drought-tolerant soil had significantly alleviated the drought stress on Arabidopsis [65]; *Mitsuaria* sp. and *Burkholderia* sp. confer the greatest drought tolerance to *Arabidopsis* and maize [23]. This suggests that application of beneficial microbiome to new host could result in a positive effect. Our results were consistent with these findings when we investigated the effects of the microbiome from non-infested soils of eggplants and cucumber on tomato plants. The galls had significantly reduced by the microbiome inoculation of the non-infested soil microbiomes. On the contrast, the galls were significantly increased when the introduction of the microbiome of the infested samples occurred.

In the past decades, plant growth promotion bacteria gained extensive attention mainly due to their beneficial effects on plant disease suppression and productivity [28]. *Pseudomonas* spp. [2, 44, 45] and *Bacillus* spp. [36, 55, 57] which belong to the phyla of Proteobacteria and Firmicutes respectively are promising candidates for the management on nematode disease through either directly antagonizing and/or inducing plant resistance to nematodes. The J2s of *M. hapla* in the most suppressive soil were found enriched with *Pseudomonas kilonensis*, which has a potentially antagonistic effect on J2s [1]. Our high-throughput sequencing analysis data revealed that non-infested soils harbored higher abundant of bacteria from Proteobacteria and Firmicutes than infested soils, indicating that there are potential plant growth promoting rhizobacteria (PGPRs) in non-infested soils. Further screening and greenhouse experiment confirmed our speculation. Two strains, characterized as *Pseudomans* sp. and *Bacillus* sp., significantly reduced the egg masses of tomato 8 weeks after J2 inoculation. There might be more undiscovered PGPRs in non-infested soil since we only screened the non-infested eggplant soil in the current study.

In our study, only culturable bacterial isolates were studied. There are still many unknowns on the unculturable microbes and their functions on RKN infection. They may not be well-characterized bacteria such as *Bacillus* and *Pseudomonas* which can directly antagonize RKN or indirectly induce plant defense. Moreover, they could have potential ecological roles in soil communities.

Application of microbiome outside of greenhouse experiment is still a challenge. Plant can modulate its rhizosphere microbiome. In addition, environmental factors such as temperature and humidity influence the soil microbiome structure [27, 53]. Our study showed that the RKN-suppressive effects were transferred from eggplant and cucumber to tomato. However, we did not analyze the newly established rhizosphere microbiome of tomato after introducing new microbiomes. It is unknown whether the newly established rhizosphere microbiome is the same as the original one and its stability after application to new host plants. The same questions may also be raised for inoculation with specific bacteria/PGPRs. Another question is transferability of microbiome’s suppressiveness to a natural field. Further experiment will be needed to test the stability of beneficial microbiome and its suppressiveness in the field.

In summary, we demonstrated that soils from non-infested areas of fields with high RKN pressure have greater microbial diversity than infested areas and inoculation of tomato roots with microbiome from non-infested soils was associated with a reduction in the number of root galls. This suggests that enrichment of the diversity and abundance of the specific microbial groups may be a way to control RKN, although it is still a challenge to manipulate the soil traits to reach the ideal community structure.

## Electronic supplementary material

Table S1 Classifications and read counts of shared OTUs and unique OTUs found in non-infested soil and infested soil. (XLSX 50 kb)

Table S2 List of common and unique taxa in non-infested and infested soils. (XLSX 36 kb)

Figure S1 Symptoms of infested and non-infested roots. Lots of galls were found in infested roots (Right); none or very few galls were observed in non-infested roots (left). (PNG 5824 kb)

High Resolution Image (TIF 9932 kb)

Figure S2 Relative sequence abundance of bacterial phyla associated with the rhizosphere soil of different plant host and infest conditions. T-test was used to compare the relative abundance’s difference between non-infested and infested soil samples in same host plant. Significant differences (*P* < 0.05) between infested and non-infested soils are indicated with lowercase letters. (PNG 365 kb)

High Resolution Image (TIF 1172 kb)

Figure S3 Neighbor-joining phylogenetic trees based on 16S rRNA and *gyrB* gene sequences of strain B1 (A) and B32 (B). (PNG 679 kb)

High Resolution Image (TIF 5104 kb)
