# Supplementary figures and images for: Rhizosphere Microbiomes from Root Knot Nematode Non-infested Plants Suppress Nematode Infection
Source: Microb Ecol. 2019 Jan 21;78(2):470–81. doi: 10.1007/s00248-019-01319-5 (PMC6657434; doi:10.1007/s00248-019-01319-5)

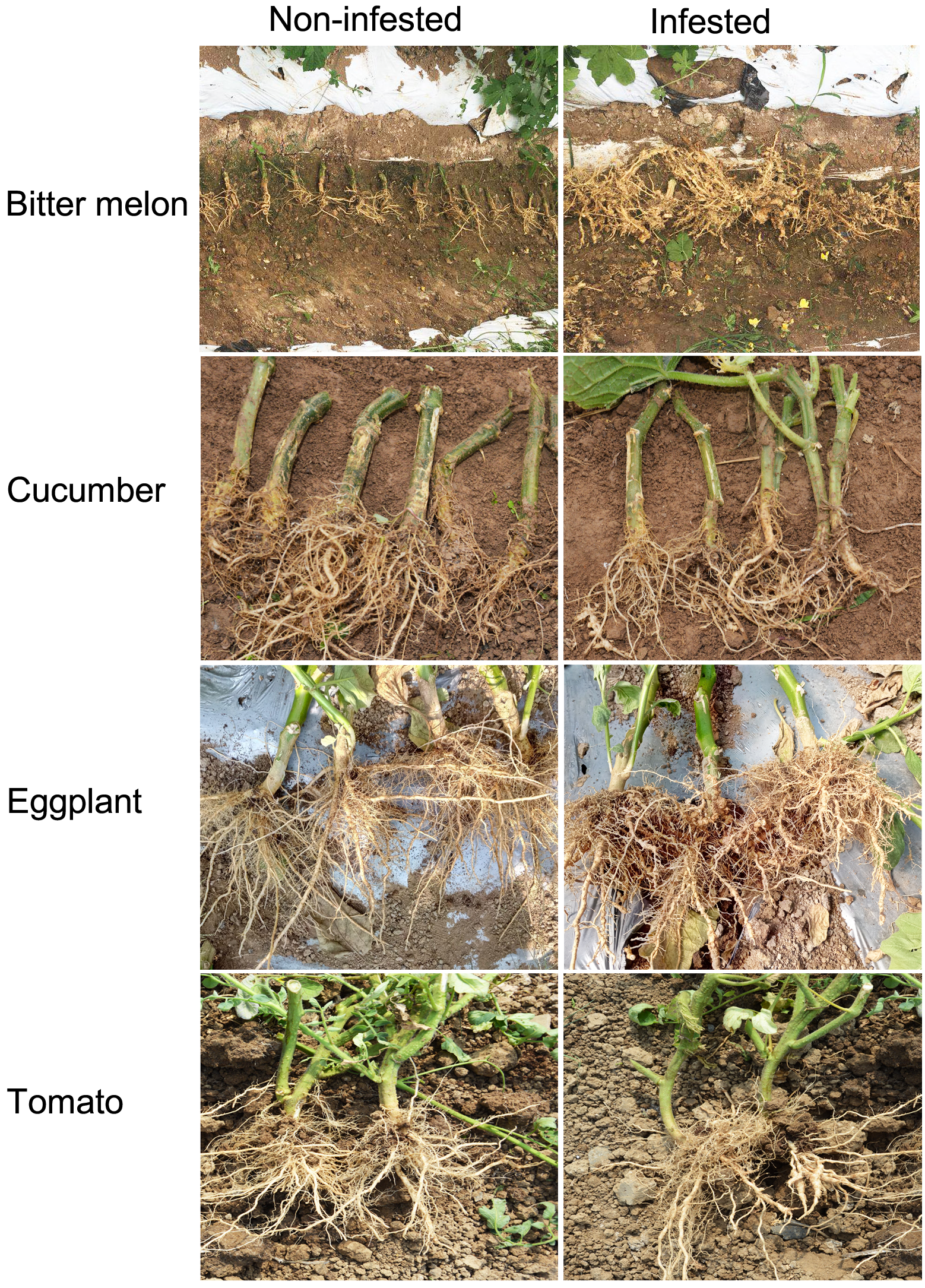

Supplement: Supplementary file 3 — Symptoms of infested and non-infested roots. Lots of galls were found in infested roots (Right); none or very few galls were observed in non-infested roots (left). (PNG 5824 kb) [file 248_2019_1319_Fig7_ESM.png]

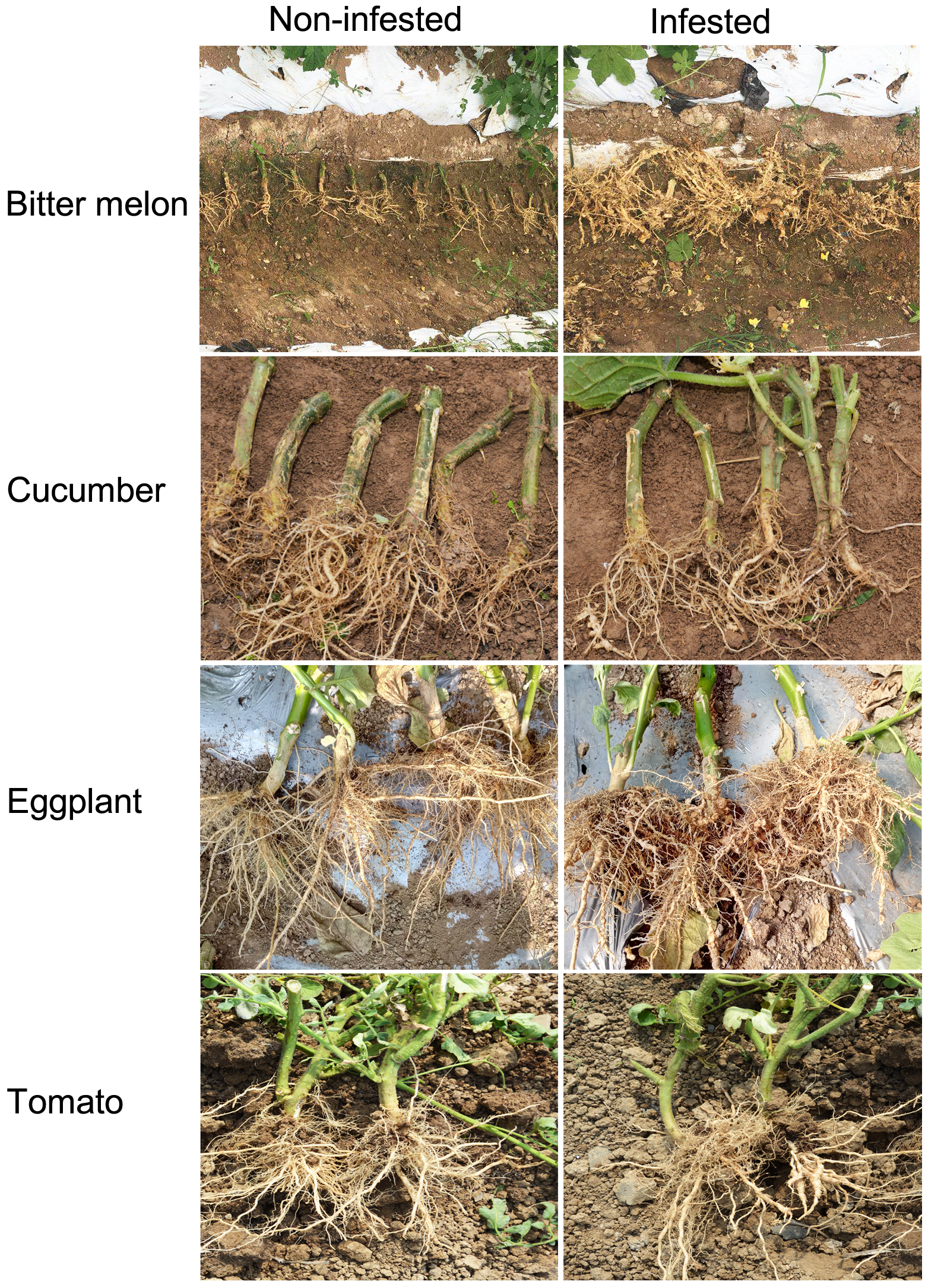

Supplement: Supplementary file 4 — High Resolution Image (TIF 9932 kb) [file 248_2019_1319_MOESM3_ESM.tif]

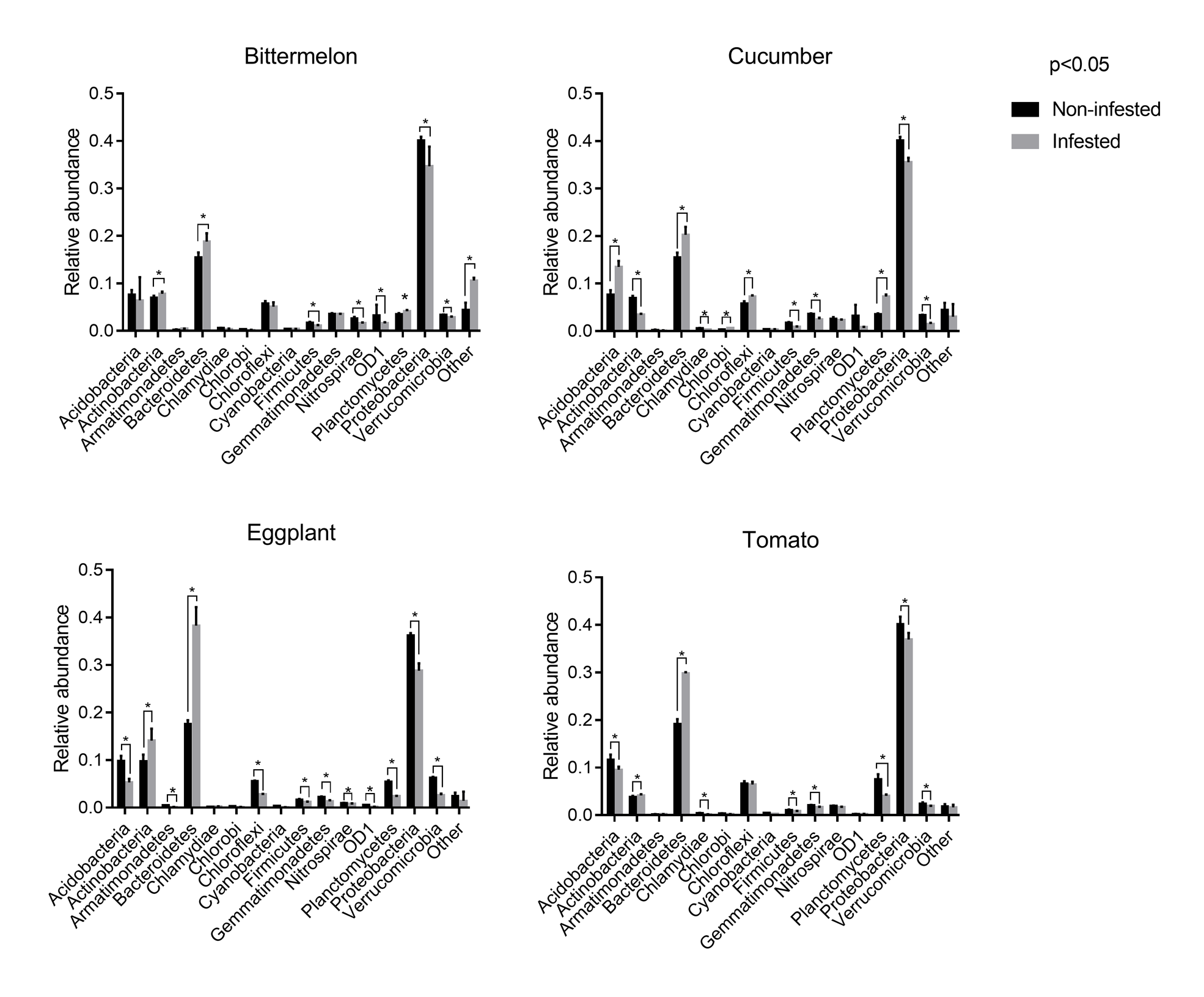

Supplement: Supplementary file 5 — Relative sequence abundance of bacterial phyla associated with the rhizosphere soil of different plant host and infest conditions. T-test was used to compare the relative abundance’s difference between non-infested and infested soil samples in same host plant. Significant differences (P < 0.05) between infested and non-infested soils are indicated with lowercase letters. (PNG 365 kb) [file 248_2019_1319_Fig8_ESM.png]

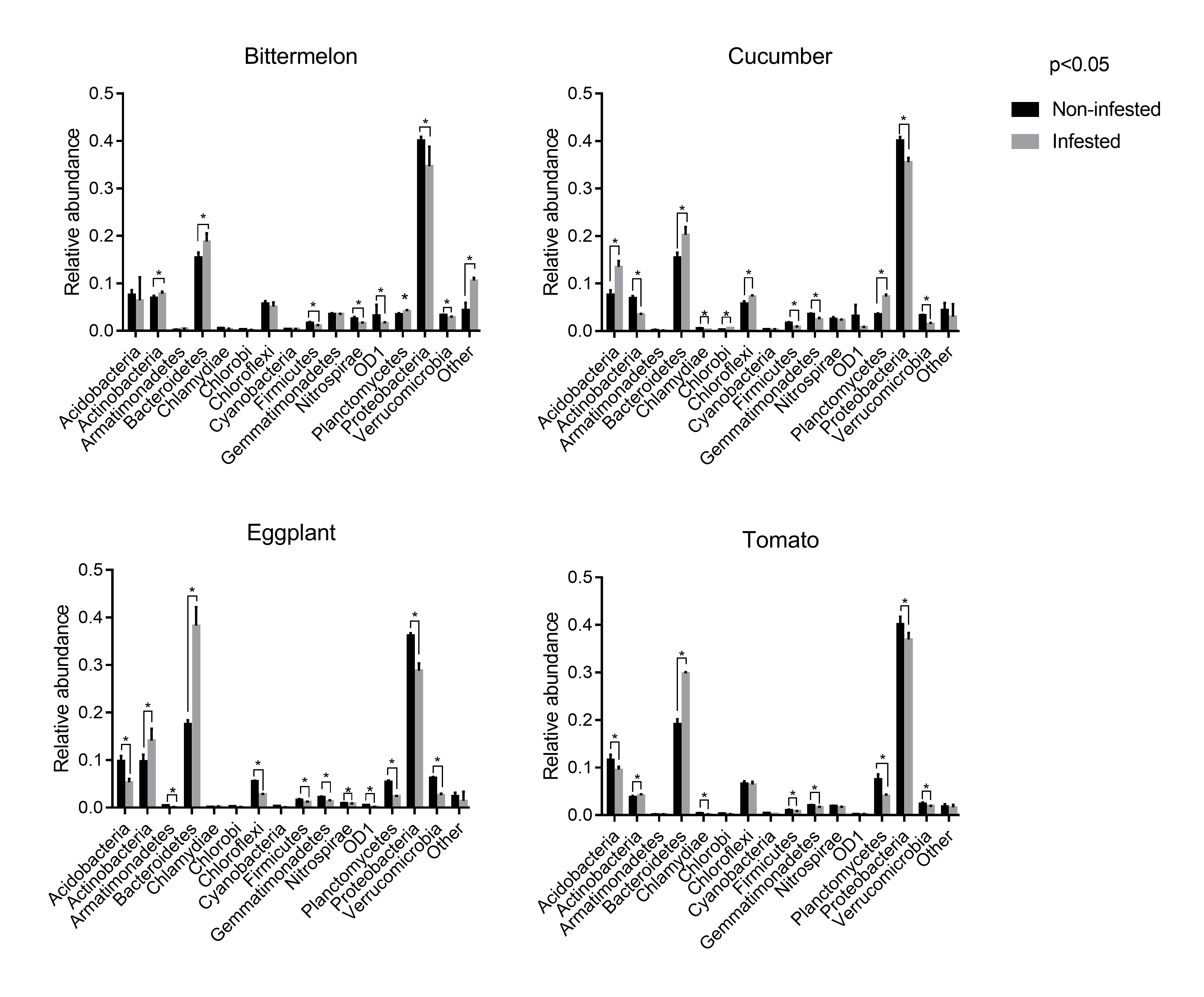

Supplement: Supplementary file 6 — High Resolution Image (TIF 1172 kb) [file 248_2019_1319_MOESM4_ESM.tif]

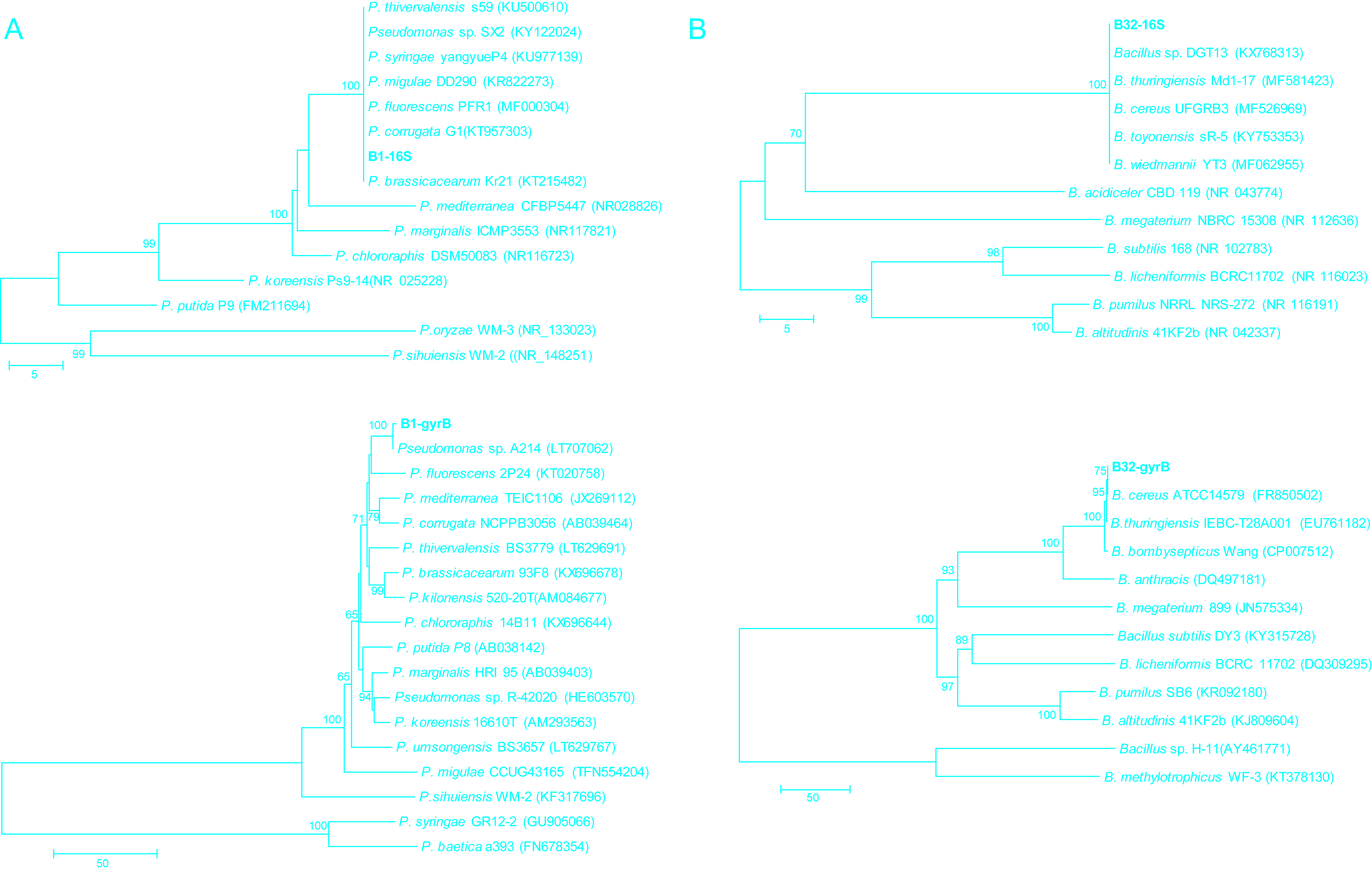

Supplement: Supplementary file 7 — Neighbor-joining phylogenetic trees based on 16S rRNA and gyrB gene sequences of strain B1 (A) and B32 (B). (PNG 679 kb) [file 248_2019_1319_Fig9_ESM.png]

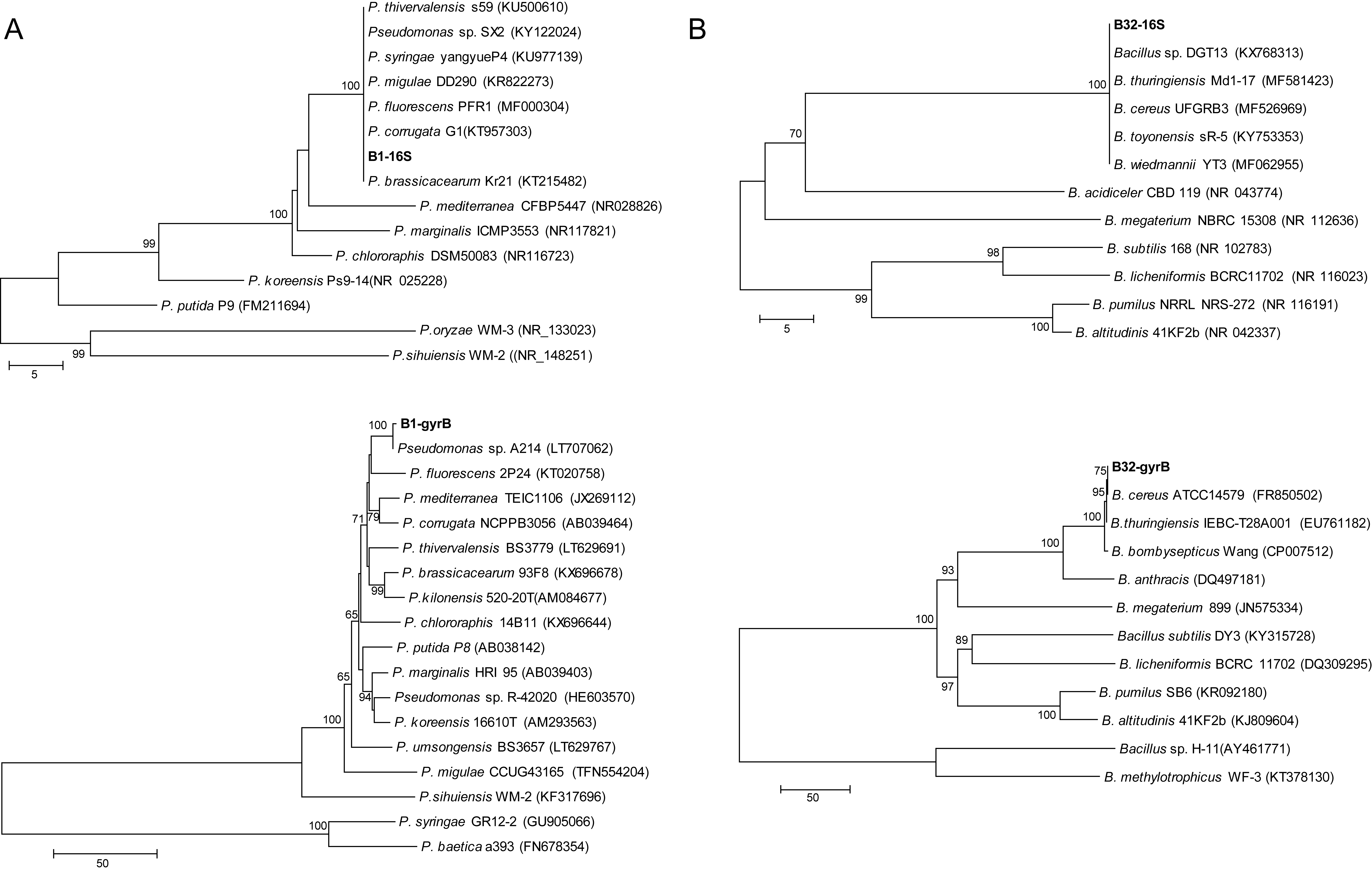

Supplement: Supplementary file 8 — High Resolution Image (TIF 5104 kb) [file 248_2019_1319_MOESM5_ESM.tif]
